# SSTR2-Targeted Theranostics in Hepatocellular Carcinoma

**DOI:** 10.3390/cancers17020162

**Published:** 2025-01-07

**Authors:** Majid Momeny, Solmaz AghaAmiri, Servando Hernandez Vargas, Belkacem Acidi, Sukhen C. Ghosh, Tyler M. Bateman, Jack T. Adams, Vahid Khalaj, Ahmed O. Kaseb, Hop S. Tran Cao, Ali Azhdarinia

**Affiliations:** 1 The Brown Foundation Institute of Molecular Medicine, McGovern Medical School, The University of Texas Health Science Center, Houston, TX 77054, USA; solmaz.aghaamiri@uth.tmc.edu (S.A.); servando.hernandezvargas@uth.tmc.edu (S.H.V.); sukhen.ghosh@uth.tmc.edu (S.C.G.); tyler.bateman@uth.tmc.edu (T.M.B.); jack.adams@uth.tmc.edu (J.T.A.); vahid.khalaj@uth.tmc.edu (V.K.); 2 Department of Surgical Oncology, Division of Surgery, The University of Texas MD Anderson Cancer Center, Houston, TX 77030, USAhstran@mdanderson.org (H.S.T.C.); 3 Department of Gastrointestinal Medical Oncology, The University of Texas MD Anderson Cancer Center, Houston, TX 77030, USA; akaseb@mdanderson.org

**Keywords:** hepatocellular carcinoma, theranostics, SSTR2

## Abstract

Background: While the clinical use of radiolabeled somatostatin analogs is well established in neuroendocrine tumors, there is growing interest in expanding their application to other somatostatin receptor 2 (SSTR2)-expressing cancers. This study investigates the potential utility of SSTR2-targeted theranostics in hepatocellular carcinoma (HCC). Methods: SSTR2 expression in HCC cell lines and clinical samples was evaluated using qRT-PCR, Western blot analysis, and a public dataset. ^67^Ga-DOTATATE uptake was measured, ^177^Lu-DOTATATE cytotoxicity was assessed, and ^68^Ga-DOTATATE tumor targeting was evaluated in HCC animal models and a patient via PET/CT imaging. Results: SSTR2 expression was confirmed in HCC cell lines and clinical samples. Radioligand uptake studies demonstrated SSTR2-mediated ^67^Ga-DOTATATE uptake. ^177^Lu-DOTATATE treatment reduced cell proliferation and enhanced the anti-tumor efficacy of the multikinase inhibitor sorafenib. ^68^Ga-DOTATATE PET/CT scans successfully identified tumors in HCC animal models and spinal metastases in a patient with HCC. Conclusion: These findings provide evidence that SSTR2-based theranostics could have significant implications for the detection and treatment of HCC.

## Simple Summary

This study explores the potential of SSTR2-targeted theranostics, integrating diagnostic and therapeutic strategies, for hepatocellular carcinoma (HCC). We confirmed substantial SSTR2 expression in both HCC cell lines and patient samples, demonstrating that radiolabeled compounds such as ^67^Ga-DOTATATE and ^177^Lu-DOTATATE—widely used for neuroendocrine tumors—can also specifically target HCC. In preclinical HCC models, these compounds exhibited effective tumor detection in vivo and significant anti-tumor activity in vitro. In a clinical case, a ^68^Ga-DOTATATE PET/CT scan revealed SSTR2 positivity in spinal metastases of an HCC patient. These results suggest that SSTR2-targeted theranostics may provide a promising, targeted approach for both the diagnosis and treatment of HCC, with the potential to improve patient outcomes in this difficult-to-treat cancer.

## 1. Introduction

Somatostatin receptor 2 (SSTR2) is a G-protein-coupled receptor that regulates key endocrine and nervous system functions by mediating the inhibitory effects of somatostatin on hormone secretion, neurotransmitter release, and cell proliferation. Under normal physiological conditions, SSTR2 is expressed in tissues such as the brain, gastrointestinal tract, and pancreas. However, in neuroendocrine tumors (NETs), SSTR2 is often significantly overexpressed, making it a highly attractive target for both diagnostic imaging and peptide receptor radionuclide therapy (PRRT) through theranostic approaches. Theranostics, which combines diagnostic precision with therapeutic efficacy, facilitates early disease detection, and targeted drug delivery, and minimizes damage to healthy tissues [1].

The overexpression of SSTR2 in NETs has enabled the development and FDA approval of radiolabeled somatostatin analogs, such as (^68^Gallium) ^68^Ga-DOTATATE for PET imaging and (^177^Lutetium) ^177^Lu-DOTATATE for PRRT, offering precise tumor visualization and effective targeted therapy [2,3]. Clinical studies have shown that SSTR2-targeted theranostics significantly improve clinical outcomes, with radiolabeled DOTATATE analogs reducing disease progression and mortality in NET patients by approximately 80% [4]. These promising results underscore the potential of SSTR2-based theranostics and support the exploration of similar approaches in other SSTR2-overexpressing malignancies.

Liver cancer is the fourth leading cause of cancer death and the sixth most diagnosed cancer globally, with over one million cases projected by 2025 [5]. In the U.S., liver cancer death rates rose by 43% from 2000 to 2016 [6]. Hepatocellular carcinoma (HCC), the most common liver cancer, has a 5-year survival rate of 18%, making it the second deadliest cancer after pancreatic cancer [6,7]. Treatment outcomes for advanced HCC remain poor, with limited benefits from conventional therapies and the anti-angiogenics like sorafenib [8,9,10]. Immunotherapies like atezolizumab are now frontline treatments [11], but drug resistance, high recurrence rates, and the lack of early detection biomarkers highlight the need for better strategies [12].

Approximately 40% of HCC samples demonstrate positive SSTR2 membrane staining, with staining intensities categorized as strong (9.6%), moderate (21.2%), and weak (7.7%) [13]. This prevalence has prompted clinical investigations into the use of somatostatin analog therapy for patients with SSTR2-positive HCC. However, findings from these studies have been inconclusive: one study reported improved survival and quality of life compared to the control group, whereas another study observed no significant enhancement in quality of life and only limited anti-cancer effects [14,15]. While the therapeutic value of somatostatin analogs in HCC remains a topic of debate, these studies underscored the utility of ^111^Indium Octreoscan scintigraphy for detecting SSTR2-positive tumors [16]. Considering the limited efficacy of non-radioactive somatostatin analogs and the promising imaging outcomes with ^111^In in HCC patients, it is notable that the modern ^68^Ga-PET/^177^Lu-PRRT theranostic paradigm has not been explored in the clinic or preclinical setting in HCC. To address this gap, we examined the efficacy of approved SSTR2-targeted radiopharmaceuticals in HCC cell lines and animal models, aiming to assess its potential as a novel imaging and therapeutic modality for this malignancy.

## 2. Materials and Methods

### 2.1. Antibodies and Chemicals

SSTR2 (clone A-8) and β-actin (clone C4) were purchased from Santa Cruz Biotechnology (Dallas, TX, USA). ^177^LuCl3 was from the National Isotope Development Center (Oak Ridge, TN, USA). ^177^Lu-DOTATATE was synthesized by mixing ^177^LuCl3 with DOTATATE, heating at 95 °C for 30 min, and cooling. Quality was checked using instant thin-layer chromatography (ITLC) and radio-HPLC. The final product was diluted with PBS according to our experimental requirement [17].

DOTATATE was radiolabeled with ^68^GaCl3 (RLS Radiopharmacies, Houston, TX, USA) or ^67^Ga citrate (Cardinal Health, Houston, TX, USA) following established methods [18]. For ^67^Ga labeling, ^67^Ga citrate was first converted to ^67^GaCl_3_ using 0.1 M HCl and subsequently purified with a Strata column. The chelation of both ^67^Ga and ^68^Ga to DOTATATE was conducted following a standardized procedure: 20 nmol of DOTATATE was mixed with ^67^Ga or ^68^Ga in 200 µL of 0.2 M NaOAc and incubated at 95 °C for 15 min with vortexing. Radiochemical yield and purity were analyzed using ITLC and radio-HPLC. The final products were subsequently diluted with PBS as needed for downstream applications.

### 2.2. Cell Culture

The SNU449, Hep3B2, HepG2, and H69 cell lines were obtained from the American Type Culture Collection, and Huh7 cells were sourced from the Japanese Collection of Research Bioresources Cell Bank. BON-1 and QGP-1 cells were provided by Dr. Jeffry A. Frost at the University of Texas Health Science Center at Houston. Cells were cultured as follows: SNU449, H69, and QGP-1 cells in RPMI supplemented with 10% FBS; Hep3B2 and HepG2 cells in DMEM with 10% FBS; and BON-1 cells in DMEM/F12 with 10% FBS. All cultures were maintained at 37 °C in a humidified incubator with 5% CO_2_, and the cells were routinely tested for mycoplasma contamination.

### 2.3. qRT-PCR, Western Blotting, and WST-1 Cell Proliferation Assay

These procedures followed previous descriptions [19]. Quantitative reverse transcription-PCR (qRT-PCR) analysis was conducted on a QuantStudio Real-Time PCR instrument (ThermoFisher, Houston, TX, USA) using PowerUp SYBR Green Master Mix (ThermoFisher, Houston, TX, USA). The primers used were *SSTR2* (F: GAGGAGCCAGGAACCCCAAA, R: GGATCCAGTGTGACATCTTTGCT) and *GAPDH* (F: TCGGAGTCAACGGATTTGGTC, R: TGAAGGGGTCATTGATGGCA). *SSTR2* expression levels were normalized to glyceraldehyde-3-phosphate dehydrogenase (*GAPDH*) levels in the same reaction. For calculations, the 2^−ΔΔCT^ formula was used, where CT is the cycle threshold.

For Western blot analysis, the cells were lysed on ice for 30 min in RIPA buffer (50 mM Tris-HCl, pH 8.0, 150 mM NaCl, 1.0% NP-40, 0.5% sodium deoxycholate, and 0.1% SDS) supplemented with protease and phosphatase inhibitors (ThermoFisher, Houston, TX, USA). The assay utilized primary antibodies and Licor secondary antibodies (Licor Biosciences, Lincoln, NE, USA), with β-actin serving as the loading control.

For the cell proliferation assay, the cells were seeded at a density of 5 × 10^3^ cells per well in 96-well plates and treated with increasing radioactivity levels of ^177^Lu-DOTATATE for 48 h. Cell viability was measured using the WST-1 assay (Sigma, Houston, TX, USA), with an untreated group serving as the control.

### 2.4. cBioPortal and the Human Protein Atlas Database

Genetic mutations and mRNA expression levels of *SSTR2* in HCC patients were analyzed using the TCGA HCC dataset [20]. The Human Protein Atlas (https://www.proteinatlas.org/), accessed on 9 January 2024, was used to investigate SSTR2 protein expression across various cancers and to examine immunohistochemistry (IHC) slides for SSTR2 expression in HCC patients.

### 2.5. Uptake Study

For radioactive uptake studies, 2 × 10^5^ cells in 96-well plates were incubated with ^67^Ga-DOTATATE (2 nM), with or without 100× octreotide block, for 1 h at 37 °C. After removing unbound radioligand, cell-associated radioactivity was quantified with a gamma counter, and the percentage of total radioactivity was calculated from a known aliquot.

### 2.6. Animal Studies

All procedures followed ethical guidelines approved by the IACUC at the University of Texas Health Science Center at Houston. Subcutaneous tumor models were created by injecting 3 × 10^6^ Huh7 or SNU449 cells into the left shoulder of athymic nude mice (n = 5/group). HCT116 colon cancer cells, which do not express SSTR2 [21], served as negative controls. Mice received an intravenous injection of 7.4 MBq (200 μCi, 2 nmol) of ^68^Ga-DOTATATE and underwent PET/CT imaging (Bruker Albira, Billerica, MA, USA) 1 h later. Tissues were then harvested for ex vivo biodistribution and radioactivity measurement.

### 2.7. Immunohistochemistry (IHC)

Tumor tissues were processed into formalin-fixed, paraffin-embedded (FFPE) blocks, which were then serially sectioned at a thickness of 5 μm. After deparaffinization, one section per block was stained with standard hematoxylin and eosin (H&E). For immunohistochemistry (IHC), sections underwent antigen retrieval followed by overnight incubation at 4 °C with an anti-SSTR2 antibody (ab134152, 1:1000, Abcam, Waltham, MA, USA). After washing with PBS, a biotinylated goat anti-rabbit polyvalent IgG secondary antibody was applied for 10 min at room temperature. Visualization was achieved using a DAB detection kit (ab64261, Abcam) according to the manufacturer’s protocol. Finally, sections were counterstained with Mayer’s hematoxylin (Fisher Healthcare, Pittsburgh, PA, USA), dehydrated through graded alcohols, cleared in xylene, and coverslipped with Cytoseal 60 mounting medium (ThermoFisher, Houston, TX, USA).

### 2.8. Statistical Analysis

Data were graphed and analyzed using GraphPad Prism Software 10.2.3 using two-way ANOVA followed by Šídák’s multiple comparisons test. The data are presented as mean ± standard deviation (SD).

## 3. Results

**Figure 1:**
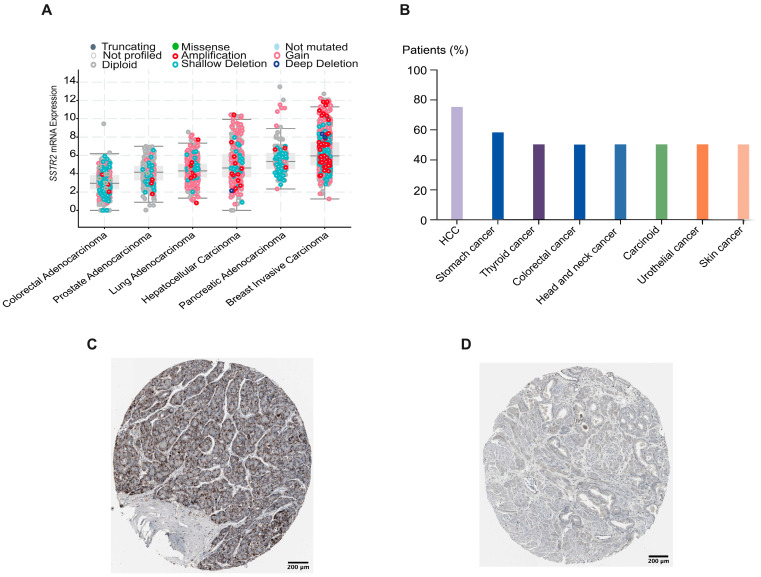
SSTR2 amplification and expression in HCC. (**A**) *SSTR2* is amplified and/or overexpressed in a significant proportion of HCC cases, based on data from the TCGA HCC dataset [20]. (**B**) Comparative analysis of SSTR2 protein expression in HCC patients versus other solid tumor types, derived from the Human Protein Atlas dataset (https://www.proteinatlas.org), accessed on 9 January 2024. (**C**,**D**) IHC staining for SSTR2 in an HCC patient (Patient ID #2766) compared with a prostate adenocarcinoma patient (Patient ID #3304), who shows negative SSTR2 staining. Images are from the Human Protein Atlas, with staining conducted using anti-SSTR2 antibody (Sigma, #HPA007264).

**Figure 2:**
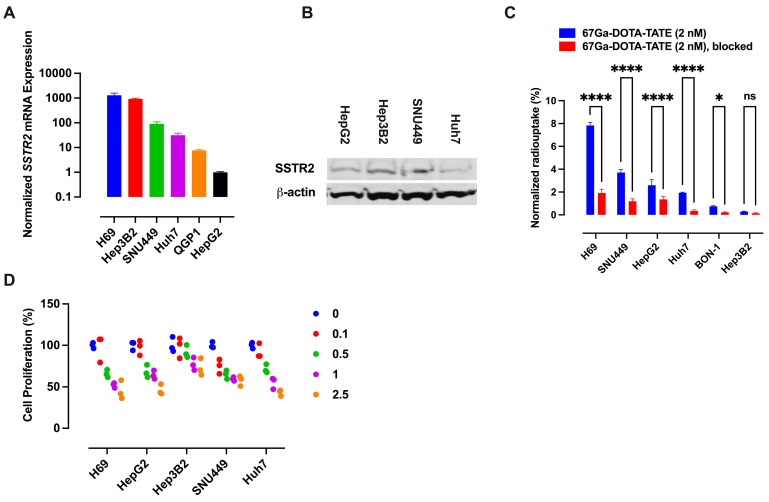
(**A**) Relative mRNA expression levels of *SSTR2* in HCC cell lines measured by qRT-PCR. Data represent the mean ± SD from three independent experiments, each conducted in triplicate. Gene expression was normalized to *GAPDH* for each cell line and further normalized to HepG2 cells. (**B**) Western blot analysis of SSTR2 protein expression in HCC cell lines, with comparison to NCI-H69 small cell lung cancer cells. β-actin was used as a loading control. (**C**) Uptake of ^67^Ga-DOTATATE in HCC cell lines relative to NCI-H69 and BON-1 cells, with receptor blocking by 100X octreotide. Data were analyzed using two-way ANOVA followed by Šídák’s multiple comparisons test, with statistical significance indicated by * *p* < 0.05 and **** *p* < 0.0001. ns indicates not significant. (**D**) Anti-proliferative effects of ^177^Lu-DOTATATE in HCC cell lines assessed by WST-1 cell proliferation assay (Sigma), with NCI-H69 cells serving as a benchmark. Data represent the mean ± SD from three independent experiments, each performed in triplicate.

The analysis of the TCGA HCC dataset [20] revealed *SSTR2* amplification or overexpression in a significant proportion of HCC patients (Figure 1A). The Human Protein Atlas dataset showed SSTR2 protein expression in most HCC patients (Figure 1B,C). We measured SSTR2 expression in HCC cell lines SNU449, Hep3B2, Huh7, and HepG2 using qRT-PCR and Western blotting, finding considerable levels of SSTR2 mRNA and protein (Figure 2A,B). Compared to NET cell lines H69 and QGP1, HCC cell lines had significant *SSTR2* mRNA levels (Figure 2A). Radioligand uptake studies confirmed substantial SSTR2-mediated ^67^Ga-DOTATATE uptake in SNU449, Huh7, and HepG2 cells, comparable to NET cell lines H69 and BON-1 (Figure 2C). To assess the anti-proliferative effects of ^177^Lu-DOTATATE, HCC cell lines were treated with increasing radioactivity for 48 h. SNU449, Huh7, and HepG2 cells showed a significant decrease in viability, while Hep3B2 cells showed minimal effects (Figure 2D).

Sorafenib, a multikinase inhibitor, is a standard first-line therapy for advanced HCC. It exerts anti-tumor effects primarily by inhibiting angiogenesis and cell proliferation through the suppression of key signaling pathways, including the RAF/MEK/ERK and VEGFR/PDGFR axes. Despite its broad molecular targets, the clinical efficacy of sorafenib remains limited. It has been shown to significantly benefit only approximately 30% of patients, with a median overall survival extension of just 2–3 months in clinical trials. Additionally, acquired resistance typically emerges within six months of treatment initiation, indicating the existence of both primary and acquired resistance mechanisms [22,23]. These challenges have prompted significant interest in strategies to enhance the anti-tumor activity of sorafenib. Combination therapies represent a promising avenue, aiming to overcome resistance mechanisms and achieve synergistic anti-cancer effects [24].

**Figure 3:**
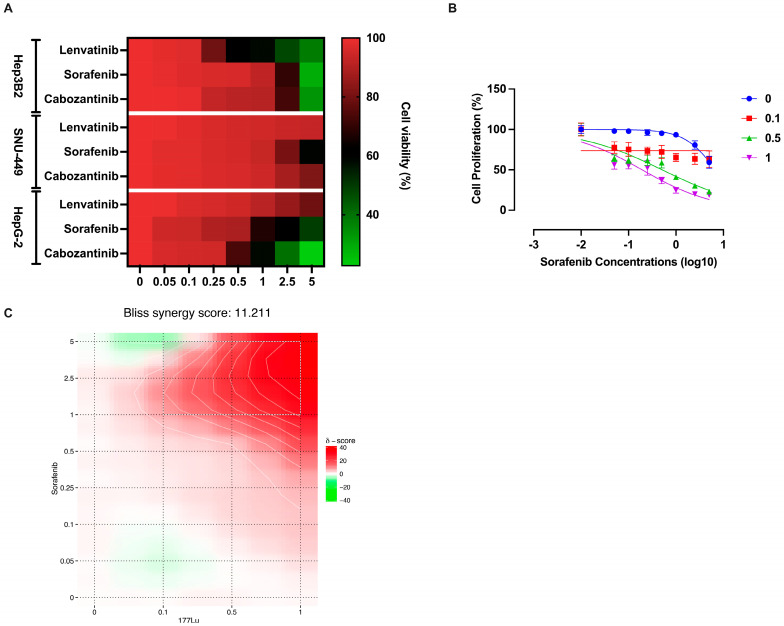
(**A**) Anti-proliferative effects of the anti-angiogenic agents lenvatinib, sorafenib, and cabozantinib on HCC cell lines. Cells were treated with increasing concentrations of these drugs (in µM) for 48 h, and cell proliferation was assessed using the WST-1 assay. (**B**,**C**) Synergistic interaction between ^177^Lu-DOTATATE and sorafenib in SNU449 cells. Co-treatment with sorafenib (0.01–5 µM) and ^177^Lu-DOTATATE (0.1, 0.5, and 1 megabecquerels (MBq)/mL) for 48 h resulted in an IC_50_ shift and synergistic effects, as determined by Bliss synergy analysis. Cell viability was measured using the WST-1 assay.

We investigated whether ^177^Lu-DOTATATE enhances sorafenib sensitivity in HCC cells. As shown in Figure 3A, the HCC cell lines exhibited resistance to clinically achievable concentrations of the anti-angiogenic agents sorafenib, lenvatinib, and cabozantinib. However, the combination of ^177^Lu-DOTATATE with sorafenib significantly increased sorafenib sensitivity in SNU449 cells. Notably, certain concentrations of the two compounds demonstrated synergistic effects, as indicated by IC_50_ shift and Bliss synergy analysis (Figure 3B,C). These findings, consistent with the observed radioligand uptake profiles, suggest that SSTR2-targeted theranostic approaches may offer a promising strategy for overcoming resistance and improving therapeutic outcomes in the management of HCC.

**Figure 4:**
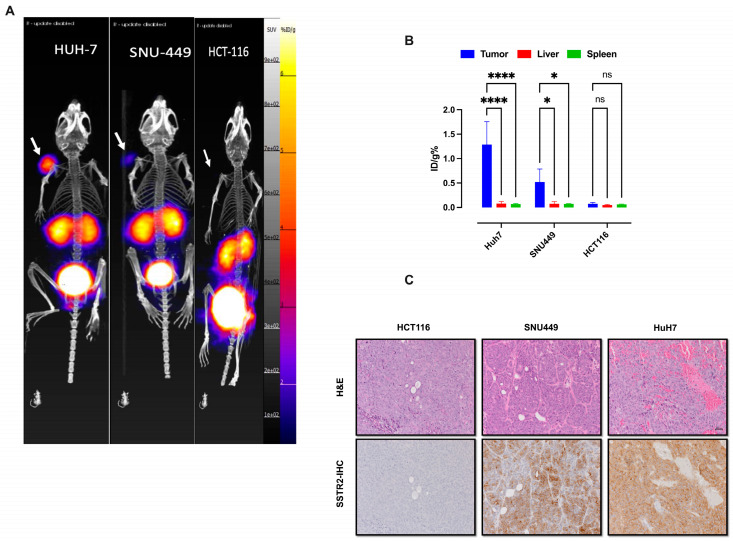
(**A**) Maximum-intensity projection showing tumor-specific ^68^Ga-DOTATATE uptake in subcutaneous models of Huh7 and SNU449. Mice were i.v. injected with 7.4 MBq of ^68^Ga-DOTATATE into the tail vein and PET/CT imaging was performed at 1 h p.i. (**B**) Quantification of radioactive biodistribution in subcutaneous models of Huh7, SNU449, and HCT116. Data were analyzed using two-way ANOVA followed by Tukey’s multiple comparisons test, with statistical significance indicated by * *p* < 0.05 and **** *p* < 0.0001. ns indicates not significant. (**C**) Corresponding IHC analysis showing SSTR2 expression in FFPE tumor sections from the mice with the HCC tumors. The tumor from the HCT116-bearing mice was used as the negative control. The scale bar is 200 μm.

To evaluate SSTR2-targeted imaging in HCC models in vivo, we used subcutaneous mouse models with Huh7 and SNU449 cells and HCT116 cells as a negative control. After IV injection of ^68^Ga-DOTATATE and PET/CT imaging, both HCC tumors were clearly visible, with radiotracer accumulation correlating with SSTR2 expression levels (Figure 4A). Ex vivo biodistribution analysis confirmed PET results, showing higher ^68^Ga-DOTATATE uptake in Huh7 and SNU449 cells compared to controls (Figure 4B). IHC analysis confirmed SSTR2 expression in tumor sections from HCC xenografts along with the absence of the target in HCT116 tumors (Figure 4C).

**Figure 5:**
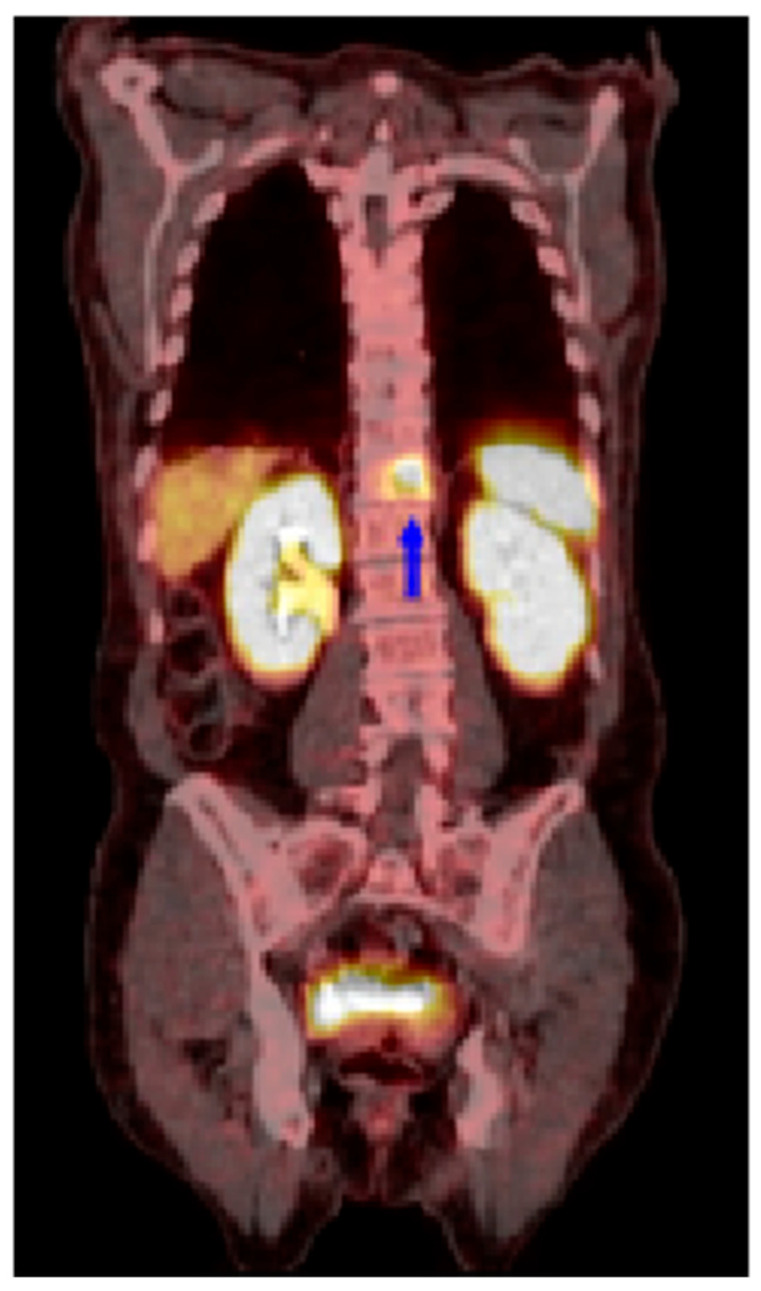
A ^68^Ga-DOTATATE PET/CT scan showing SSTR2 positivity in spinal metastases from a patient with HCC. The blue arrow indicates the location of the spinal metastases originating from the liver.

Notably, a ^68^Ga-DOTATATE PET/CT scan conducted on a patient with a history of HCC demonstrated SSTR2 positivity in spinal metastases (Figure 5). The patient had previously undergone definitive radiation therapy for HCC and later required a small bowel resection. During routine surveillance for NET, a ^68^Ga-DOTATATE PET/CT scan detected a tumor in the spine. Initially suspected to be a metastasis from the NET, the biopsy of the lesion confirmed it was HCC. Collectively, these novel findings illustrate the roles of SSTR2 as a functional biomarker for theranostic-based detection and treatment in HCC and emphasize the need for further clinical investigations.

## 4. Discussion

There is growing interest in applying SSTR2-based theranostics to malignancies beyond NETs. A 2017 study on breast cancer tested the binding of ^111^In-DOTA-Tyr^3^-octreotate (an SSTR agonist) and ^111^In-DOTA-JR11 (an SSTR antagonist) to 40 human breast specimens using in vitro autoradiography, revealing that SSTR antagonists hold promise for breast cancer imaging [25]. Further support comes from a more recent study where preclinical microarrays indicated that 51% of ER-positive and 18% of ER-negative breast cancer samples were enriched for SSTR2. In a phase 2 trial with 30 participants with metastatic ER-positive breast cancer, DOTATATE PET/CT identified strong SSTR2 expression in 30% of cases, with one patient showing a near complete response to ^225^Actinium-DOTATATE therapy. These findings suggest that SSTR2-targeted molecular imaging and radioligand therapy offer a novel therapeutic option for patients with metastatic breast cancer [22].

To further expand the application of SSTR2-targeted theranostics, we performed novel investigation in the context of HCC. The examination of SSTR2 expression in HCC cell lines and clinical specimens revealed notable uptake of ^67^Ga-DOTATATE in 75% of examined HCC cell lines. Subsequent treatment with ^177^Lu-DOTATATE resulted in a significant reduction in cell viability and increased the potency of the multi-kinase inhibitor sorafenib, suggesting potential utility of this approach for treating HCC. We also showed that ^68^Ga-DOTATATE PET/CT imaging can effectively localize HCC in subcutaneous tumor models with varying SSTR2 expression levels and an HCC patient with spinal metastases. This observation, in combination with the low background signal from PET and ex vivo biodistribution analysis, indicates the feasibility of tumor delineation in patients with HCC. Our results present strong evidence for the feasibility and the potential diagnostic visualization and therapeutic impact of SSTR2-targeted theranostics in the management of HCC, warranting further clinical exploration.

## 5. Conclusions

In conclusion, our study demonstrates the potential of SSTR2-targeted theranostics for HCC. We observed significant ^67^Ga-DOTATATE uptake in 75% of HCC cell lines and reduced cell viability with ^177^Lu-DOTATATE treatment. Additionally, ^68^Ga-DOTATATE PET/CT successfully localized tumors in models with different SSTR2 expressions and an HCC patient. These findings support further clinical exploration of SSTR2 theranostics in HCC management.

## Data Availability

Data from this study are available upon reasonable request from the corresponding authors.

## References

[1] Palekar-Shanbhag P., Jog S.V., Chogale M.M., Gaikwad S.S. (2013). Theranostics for cancer therapy. Curr. Drug Deliv..

[2] Reubi J.C., Waser B., Schaer J.C., Laissue J.A. (2001). Somatostatin receptor sst1-sst5 expression in normal and neoplastic human tissues using receptor autoradiography with subtype-selective ligands. Eur. J. Nucl. Med..

[3] Ginj M., Zhang H., Waser B., Cescato R., Wild D., Wang X., Erchegyi J., Rivier J., Macke H.R., Reubi J.C. (2006). Radiolabeled somatostatin receptor antagonists are preferable to agonists for in vivo peptide receptor targeting of tumors. Proc. Natl. Acad. Sci. USA.

[4] Strosberg J., El-Haddad G., Wolin E., Hendifar A., Yao J., Chasen B., Mittra E., Kunz P.L., Kulke M.H., Jacene H. (2017). Phase 3 Trial of (177)Lu-Dotatate for Midgut Neuroendocrine Tumors. N. Engl. J. Med..

[5] Villanueva A. (2019). Hepatocellular Carcinoma. N. Engl. J. Med..

[6] Craig A.J., von Felden J., Garcia-Lezana T., Sarcognato S., Villanueva A. (2020). Tumour evolution in hepatocellular carcinoma. Nat. Rev. Gastroenterol. Hepatol..

[7] Sung H., Ferlay J., Siegel R.L., Laversanne M., Soerjomataram I., Jemal A., Bray F. (2021). Global Cancer Statistics 2020: GLOBOCAN Estimates of Incidence and Mortality Worldwide for 36 Cancers in 185 Countries. CA Cancer J. Clin..

[8] Forner A., Reig M., Bruix J. (2018). Hepatocellular carcinoma. Lancet.

[9] Forner A., Llovet J.M., Bruix J. (2012). Hepatocellular carcinoma. Lancet.

[10] Ladd A.D., Duarte S., Sahin I., Zarrinpar A. (2023). Mechanisms of drug resistance in HCC. Hepatology.

[11] Storandt M.H., Zemla T.J., Patell K., Naleid N., Gile J.J., Tran N.H., Chakrabarti S., Jin Z., Borad M., Mahipal A. (2024). Atezolizumab plus bevacizumab as first-line systemic therapy for hepatocellular carcinoma: A multi-institutional cohort study. Oncologist.

[12] Gerbes A., Zoulim F., Tilg H., Dufour J.F., Bruix J., Paradis V., Salem R., Peck-Radosavljevic M., Galle P.R., Greten T.F. (2018). Gut roundtable meeting paper: Selected recent advances in hepatocellular carcinoma. Gut.

[13] Lequoy M., Desbois-Mouthon C., Wendum D., Gupta V., Blachon J.L., Scatton O., Dumont S., Bonnemaire M., Schmidlin F., Rosmorduc O. (2017). Somatostatin receptors in resected hepatocellular carcinoma: Status and correlation with markers of poor prognosis. Histopathology.

[14] Kouroumalis E., Skordilis P., Thermos K., Vasilaki A., Moschandrea J., Manousos O.N. (1998). Treatment of hepatocellular carcinoma with octreotide: A randomised controlled study. Gut.

[15] Cebon J., Findlay M., Hargreaves C., Stockler M., Thompson P., Boyer M., Roberts S., Poon A., Scott A.M., Kalff V. (2006). Somatostatin receptor expression, tumour response, and quality of life in patients with advanced hepatocellular carcinoma treated with long-acting octreotide. Br. J. Cancer.

[16] Dimitroulopoulos D., Xinopoulos D., Tsamakidis K., Zisimopoulos A., Andriotis E., Markidou S., Panagiotakos D., Chrysohoou C., Bazinis A., Paraskevas E. (2002). The role of sandostatin LAR in treating patients with advanced hepatocellular cancer. Hepatogastroenterology.

[17] Nicolas G.P., Mansi R., McDougall L., Kaufmann J., Bouterfa H., Wild D., Fani M. (2017). Biodistribution, Pharmacokinetics, and Dosimetry of (177)Lu-, (90)Y-, and (111)In-Labeled Somatostatin Receptor Antagonist OPS201 in Comparison to the Agonist (177)Lu-DOTATATE: The Mass Effect. J. Nucl. Med..

[18] Ghosh S.C., Hernandez Vargas S., Rodriguez M., Kossatz S., Voss J., Carmon K.S., Reiner T., Schonbrunn A., Azhdarinia A. (2017). Synthesis of a Fluorescently Labeled (68)Ga-DOTA-TOC Analog for Somatostatin Receptor Targeting. ACS Med. Chem. Lett..

[19] Momeny M., Tienhaara M., Sharma M., Chakroborty D., Varjus R., Takala I., Merisaari J., Padzik A., Vogt A., Paatero I. (2024). DUSP6 inhibition overcomes neuregulin/HER3-driven therapy tolerance in HER2+ breast cancer. EMBO Mol. Med..

[20] Cerami E., Gao J., Dogrusoz U., Gross B.E., Sumer S.O., Aksoy B.A., Jacobsen A., Byrne C.J., Heuer M.L., Larsson E. (2012). The cBio cancer genomics portal: An open platform for exploring multidimensional cancer genomics data. Cancer Discov..

[21] Hernandez Vargas S., Kossatz S., Voss J., Ghosh S.C., Tran Cao H.S., Simien J., Reiner T., Dhingra S., Fisher W.E., Azhdarinia A. (2019). Specific Targeting of Somatostatin Receptor Subtype-2 for Fluorescence-Guided Surgery. Clin. Cancer Res..

[22] Cheng A.L., Kang Y.K., Chen Z., Tsao C.J., Qin S., Kim J.S., Luo R., Feng J., Ye S., Yang T.S. (2009). Efficacy and safety of sorafenib in patients in the Asia-Pacific region with advanced hepatocellular carcinoma: A phase III randomised, double-blind, placebo-controlled trial. Lancet Oncol..

[23] Llovet J.M., Ricci S., Mazzaferro V., Hilgard P., Gane E., Blanc J.F., de Oliveira A.C., Santoro A., Raoul J.L., Forner A. (2008). Sorafenib in advanced hepatocellular carcinoma. N. Engl. J. Med..

[24] Kudo M. (2018). Combination Cancer Immunotherapy in Hepatocellular Carcinoma. Liver Cancer.

[25] Dalm S.U., Haeck J., Doeswijk G.N., de Blois E., de Jong M., van Deurzen C.H.M. (2017). SSTR-Mediated Imaging in Breast Cancer: Is There a Role for Radiolabeled Somatostatin Receptor Antagonists?. J. Nucl. Med..

